# Plant-Derived Hydrolysates Are a Suitable Replacement for Tryptone N1 in Recombinant Protein Expression Using Human Embryonic Kidney (HEK293-6E) Cells

**DOI:** 10.3390/biotech15010014

**Published:** 2026-02-05

**Authors:** Shafqat Shabir, Md. Shahadat Hossain, Lucie Egly, Gizem Yalkin, Franco H. Falcone

**Affiliations:** 1 Institute of Parasitology, Justus-Liebig-University Giessen, 35392 Giessen, Germany; shafqat.shabir@vetmed.uni-giessen.de (S.S.); shahadat.para@bau.edu.bd (M.S.H.); 2 Department of Parasitology, Bangladesh Agricultural University, Mymensingh 2202, Bangladesh; 3 Spécialité Génie Biologique et Santé, Polytech Angers, 49000 Angers, France; egly.lucie@gmail.com; 4 Organotechnie, 93120 La Courneuve, France; g.yalkin@organotechnie.com

**Keywords:** HEK293, recombinant expression, hydrolysates, Tryptone N1, plant-derived

## Abstract

Human embryonic kidney (HEK293) cells are a widespread choice for recombinant protein expression. To optimise yields, the hydrolysate Tryptone N1 (TN1) is commonly added post-transfection. TN1 is obtained by controlled enzymatic digestion of casein. As an animal by-product, TN1 faces stricter regulations during cross-country shipments than plant-based products. This raises the question of whether plant-derived peptides are a suitable alternative to TN1. Using polyethyleneimine (PEI) as a cationic polymer, we transfected HEK293-6E cells grown in suspension in serum-free medium and divided the transfectants into four groups (each in triplicate). Two plant-based hydrolysates each derived from pea and broad bean were compared with TN1 and a no-hydrolysate control group. We monitored the cultures for total cell numbers and viability at days 1, 4, and 5 post-transfection. Both plant-based hydrolysates and TN1 showed similar live cell percentages, in contrast to the no-hydrolysate control, which showed lower viability. Five days post-transfection, the expressed His-tagged protein, a tegumental antigen from the eukaryotic parasite *Echinococcus granulosus*, was retrieved from the serum-free culture supernatant, and the expressed recombinant protein was quantified. The linear ranges for the protein load on the stain-free blot and for the use of the fluorescent anti-His-Tag Alexa_488_ antibody were determined. Using these parameters, stain-free Western blotting and total protein normalization were performed. The plant-derived pea and broad bean hydrolysates reproducibly resulted in similar expression levels as animal-derived TN1; all three hydrolysates were better than no hydrolysate. We conclude that plant-derived hydrolysates are a suitable, more sustainable replacement for TN1.

## 1. Introduction

Recombinant expression of proteins in prokaryotic and yeast expression systems is characterised by high expression levels [1]. However, many proteins also require specific post-translational modifications (PTMs) for stability, structure, and biological activity [2]. In such cases, mammalian expression systems such as human embryonic kidney (HEK293) cells or Chinese Hamster Ovary (CHO) cells are a suitable option, as these can carry out the necessary PTMs [3].

Stable transfections in mammalian expression systems for PTM-bearing proteins require lengthy selection procedures; they are resource-intensive, time-consuming, and can still lead to variable expression [4]. Transient transfection is seen as a better alternative because it is fast, flexible, and cost-effective, but can be problematic when low yields are achieved [5]. To improve yields, different approaches have been tested, such as increasing the transfection efficiency [6,7] or increasing the metabolic profiles of transfectants after transfection [8]. However, a high transfection efficiency cannot overcome intrinsic limitations such as pools of transcription factors, tRNA, and RNA polymerases, meaning that only a limited quantity of mRNA can be synthesised [9]. Therefore, a high transfection efficiency can also result in toxicity [10]. Toxicity can be reduced by the choice of appropriate transfection reagents, for example, as shown by Almulathanon and colleagues [11] by favouring linear polyamidoamine over branched polyethyleneimine polyplexes, which resulted in a lower transfection efficiency, but significantly less cytotoxicity. After transfection, several additions or conditions can be applied to boost expression of the target protein. For example, lower temperatures (mild hypothermia, 31 °C instead of 37 °C) have been shown to increase recombinant protein yields in CHO cells, leading to yields as high as 60–80 mg/L after transient transfection [12]. Although the addition of serum also increases the yield of recombinant protein, its interference with downstream purification makes it less desirable, along with issues such as cost and scalability. Therefore, widely used protocols use serum-free media supplemented with hydrolysates consisting of low-molecular-weight peptides and other components derived from controlled proteolysis. It has been shown that not only the composition but also the time of addition of these hydrolysates have positive effects on the expression [8,13]. The addition of specific combinations of yeast hydrolysates has also been shown to increase the expression by up to 180% [14]. Work by Schlaeger has shown that the addition of hydrolysates can reduce the rates of apoptosis [15]. The nutritional benefits of addition of peptones have also been observed in the BHK-21 cell line [16].

Among eukaryotic expression systems, human embryonic kidney (HEK293) cells are a widely used expression system because of their PTMs, ease of transfection with cationic polymers, and the option of scalability [2]. HEK293 cells can be adapted to grow in suspension, allowing higher cell densities and yields. To increase production, various factors, such as the quantity of DNA [17]; the addition of sodium acetate [18], sodium butyrate, valproic acid, and dimethyl sulfoxide [19]; and higher cell density [20], have been studied. Valproic acid has been shown to act as a histone deacetylase inhibitor [21], preventing transcriptional silencing of genes, and to increase yields by inducing cell cycle arrest in the G2/M phase while prolonging survival of CHO cells in culture [22].

Free amino acids and oligopeptides derived from protein hydrolysates do not use the same transport system but rather different, specialized transport systems. Not much is known about transport systems on CHO or HEK293 cells, as these do not express ‘classic’ proton-coupled oligopeptide transporters such as PEPT1 (SLC15A1) or PEPT2 (SLC15A2), which are restricted to the intestinal epithelium and the kidney, for reabsorption of nutritional nitrogen [23]. In fact, when used for transport studies, such cells need to be transfected to overexpress peptide transport systems, as the endogenous expression is very low [24]. Work by Bonarius and coauthors using metabolic flux analysis suggests that growth and antibody production in a hybridoma cell line were increased when using meat-derived hydrolysates (Primatone RL) as the supplement rather than amino acids, which is perhaps explained in part by the more energy-efficient peptide transport into the cell compared with the transport of free amino acids [25].

Once inside the cells, oligopeptides are further processed by different peptidases. The resulting amino acids can either be taken up by the Tricarboxylic acid (TCA) cycle (after deamination or transamination) or be used in protein synthesis [16]. Therefore, hydrolysate supplements added during expression, rather than the corresponding free amino acid mixtures, are considered a necessary step for improved yield in HEK293 cell expression [26,27]. The most commonly used hydrolysate is Tryptone N1 (TN1), which is obtained by the controlled enzymatic proteolysis of casein protein [26,28].

An alternative to animal-derived hydrolysates is the use of plant-derived hydrolysates. Avoidance of animal-derived proteins conveniently eliminates potential sources of mycoplasma or viral contamination [16]. Furthermore, the transmission of notifiable animal diseases from endemic countries to non-endemic areas is a growing concern, which limits the use of animal-derived products for research purposes. This obstacle and the increasing demand for more sustainable products and processes underpin the need for plant-based alternatives. However, such options need to be thoroughly validated to replace the standard supplements. In the current study, we compared TN1 with novel, not yet commercialised broad bean-derived (F120) and pea-derived (P112) hydrolysates in terms of their ability to support post-transfection growth and recombinant protein yields.

## 2. Materials and Methods

### 2.1. HEK293-6E Cell Culture

HEK293-6E cells (expression platform licensed from the National Research Council Canada, NRC files 11266 and 11565) were maintained in culture in a 100 mL flask on a rotating platform at 120 rpm at 37 °C with 5% CO_2_ in a humidified incubator. Cell viability was assessed using a TC20 automated cell counter (Bio-Rad, Feldkirchen, Germany) with 0.4% Trypan Blue, as instructed by the manufacturer.

### 2.2. HEK293 Cell Transfection

Cells were diluted to 1 × 10^6^ cells/mL before the day of transfection in 1 L flasks containing 300 mL of FreeStyle F17 serum-free medium (Invitrogen, Thermo Fisher Scientific, Dreieich, Germany) [28]. On the day of transfection, the cell density and viability were measured in triplicate. All transfections were carried out at 1.8 ± 0.1 × 10^6^ cells/mL and viability > 97% [28]. For transfection, 7.5 mL of pre-warmed medium was taken in two 15 mL tubes and used to resuspend 600 µg of PEI MAX^®^ (Linear Polyethyleneimine, MW 40 kDa, Polysciences, Washington, PA, USA) and 300 µg of plasmid, respectively. Two plasmids were used in this study. The GFP-encoding plasmid pTTo-GFPq was used as a control to confirm successful transfection and to monitor the reagent quality. Protein expression was carried out using the cDNA sequence encoding an *Echinococcus granulosus* tegumental antigen (UniProt ID: U6JFD4) fused with a C-terminal Myc and His-8 tag encoded in pTT28, which also includes a strong secretory signal. Both DNA and PEI solutions were vortexed and mixed in a 50 mL tube. The mixture was also vortexed 3 times for 1 s each and incubated at room temperature for 3 min. The DNA–PEI mixture was slowly added to 300 mL medium [28]. In parallel, a 2 mL transfection was performed with the GFP plasmid to monitor the transfection success. Pictures were taken 24 h after transfection on a Zeiss Primovert inverted microscope (ZEISS, Jena, Germany) equipped with a Zeiss Axiocam 208 colour digital camera (ZEISS, Jena, Germany) in the GFP channel.

### 2.3. Hydrolysate Feeding

Twenty-four hours after transfection, the cells were removed from the 1 litre flask and split into 12 new 125 mL flasks after thorough but careful mixing (for the workflow, see Supplementary Figure S1). This step ensured that all flasks were seeded with the same batch of transfected cells, ensuring equal transfection efficiency for all technical repeats and feeding conditions. Each flask received 25 mL of transfected cell suspension. All hydrolysates were from Organotechnie (La Courneuve, France). Hydrolysate stock solutions 20% (*w*/*v*) of TN1, P112, and F-120 were prepared in HEK293 Free Style F17 cell medium, filtered with a 0.2 µm membrane. Each hydrolysate was added to flasks to a final concentration of 0.5% *w*/*v* (3 technical replicates); 3 flasks were not fed any hydrolysate as a negative control. All 12 flasks were kept on a shaking platform in a humidified incubator with 5% CO_2_. Before the addition of hydrolysate (day 1 post-transfection) and on days 4 (96 h) and 5 (120 h), an aliquot from each flask was taken out for cell density and viability measurements. Two readings (R1, R2) were taken for each condition and averaged.

### 2.4. Harvest

On day 5 after transfection, the HEK cell-expressed protein was harvested. Supernatants from all 12 flasks were transferred to separate 50 mL tubes and centrifuged at 3200× *g* for 30 min. The supernatant was collected and filtered with a 0.2 µm syringe filter.

### 2.5. Trichloroacetic Acid (TCA) Protein Precipitation

A total of 300 µL of TCA was mixed with 1200 µL of protein sample from filtered supernatants of all 12 flasks. The mixture was incubated for 10 min on ice. After centrifugation at 14,000 rpm, the supernatant was removed, and the pellet was washed with 200 µL of cold acetone and centrifuged at 14 K rpm twice. The pellet was then dried at 95 °C for 5 min in a heat block. Pellets were then dissolved in 30 µL of 4× SDS-PAGE reducing loading buffer and boiled for 10 min before loading onto Criterion™ TGX Stain-Free™ Protein Gel, 4–15% (Bio-Rad).

### 2.6. Determination of Combined Linear Dynamic Range and Quantification

For quantitative Western blotting, saturated blots can underestimate the differences in protein amounts; therefore, the linear dynamic range of our protein, detected with a fluorescently labelled antibody to the His-Tag, was measured. Protein precipitates from all 12 tubes were pooled in a single tube [29]. A two-fold serial dilution was prepared and loaded into Criterion™ TGX Stain-Free™ Protein Gels, 4–15% (Bio-Rad) to produce a standard curve. Following protein separation at 150 V for 1 h, the gel image (stain-free) was taken using a ChemiDoc MP imaging system (Bio-Rad, Feldkirchen, Germany). The protein in the gels was transferred using the Trans-Blot Turbo RTA Midi 0.2 µm PVDF Transfer Kit (Bio-Rad) onto a methanol-activated PVDF membrane. The stain-free blot image was also taken before blocking. After blocking, the membrane was incubated with anti-His-tag-Alexa_488_ antibody at 1:1000 overnight at 4 °C. The next morning, the membrane was washed 6 times (3–5 min each) with TBST buffer, and an image was taken in ChemiDoc [30]. Protein was quantified by densitometry analysis of the Western blots. Membrane images from blotted stain-free gels or stained with an Alexa_488_-conjugated anti-His-tag antibody were taken and analysed in Image Lab software (version 6.1, Bio-Rad). The band intensities were normalised against the total protein on the stain-free blot according to [29]. A graph was developed against the loaded dilution series and band intensity. After obtaining the linear dynamic range for protein loading, a separate Western blotting experiment was performed to determine the antibody dilution factor. For this, the blot was developed by loading a 1/8 dilution of protein, as determined by previous experiments. The blot was cut into eight strips, each containing the same amount of protein [31]. The strips were incubated with different antibody dilutions ranging from 1:250 to 1:16,000. After determining the linear range for protein loading and antibody dilutions, three Western blots for quantifying expressed proteins across three biological replicates were developed. For each experiment, the normalised volume intensities of all 12 samples were recorded. To obtain the n-fold relative difference, each value was divided by the average value of three TN1 technical replicates.

### 2.7. Statistical Analysis

All data were analysed using GraphPad Prism (version 8.0.2, GraphPad Software). The average of the calculated fold-difference values of protein expression of 3 technical replicates of TN1, P112, F112, or no hydrolysate control from 3 independent biological repeats was taken. Data were analysed with one-way ANOVA and Tukey’s multiple comparison test.

## 3. Results

**Figure 1:**
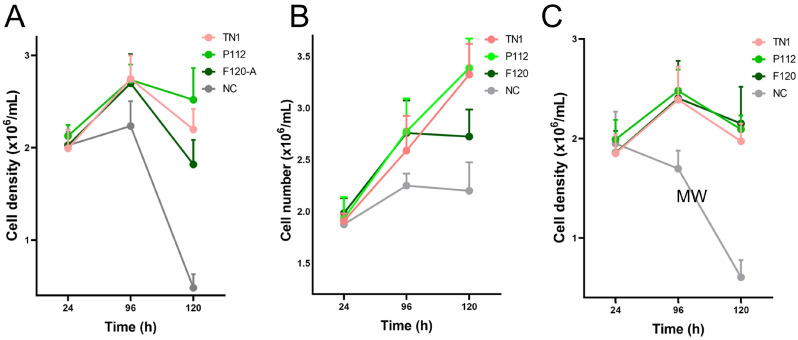
Viable cell counts of HEK293-6E cells from 24 to 120 hpt in three independent experiments (N = 3, biological replicates). Each biological replicate (**A**–**C**) was performed in triplicate technical replicates (n = 3), with two cell counts averaged for each sample at each time point.

**Figure 2:**
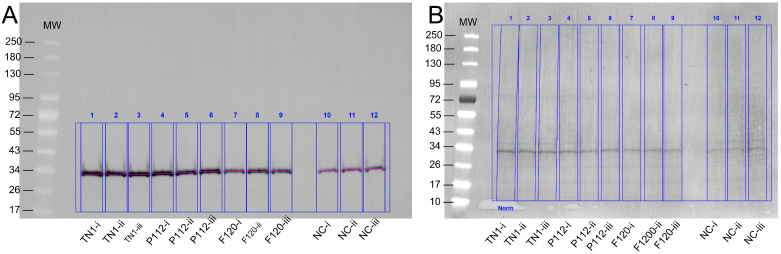
(**A**) Example of quantification of expressed protein. The Western blot was developed with an anti-His-tag Alexa_488_ fluorescent antibody. Three technical replicates of each hydrolysate are denoted as i, ii, and iii, while NC is the negative control. The band appeared around the expected size of 30–32 kDa. (**B**) Stain-free blot image used for total lane protein normalisation.

**Figure 3:**
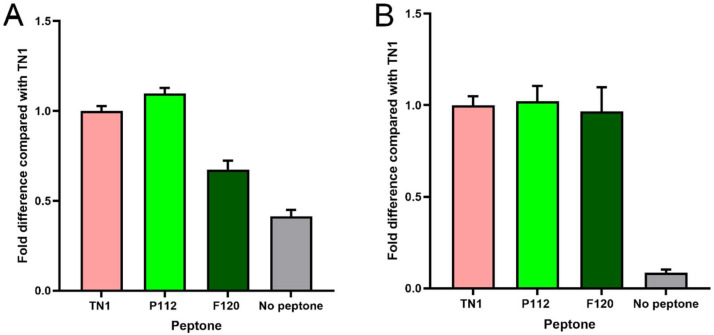

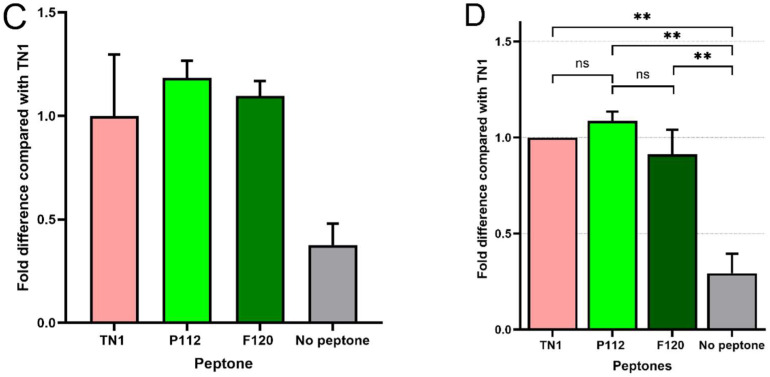
Comparison of protein expression as a fold difference relative to TN1. (**A**–**C**) Data of the 3 independent experiments shown individually, where the average of three technical replicates in each experiment is shown with standard deviations. (**D**) Data from each biological replicate combined in a single graph compared with TN1 (N = 3, n = 3). ** indicates *p* < 0.01, ns: not significant, using one-way ANOVA.

The transfection efficiency was monitored by transfection of a GFP-encoding plasmid in parallel. The overall transfection efficiency was similar (about 30%). However, all transfected cells were equally split into 12 individual flasks for each of the three experiments. This rules out differences in the transfection efficiency as a random source of variation between the experimental repeats. Twenty-four hours post-transfection (hpt), the live cell counts and live cell percentages were measured. As shown in Figure 1, hydrolysate-fed triplicates showed higher viability than transfectants without hydrolysate addition (all cell counts are shown in Supplementary Table S1). Two viability cell counts were taken from each flask during the culture at 24, 96, and 120 hpt. We observed that the total number of live cells in the supplemented groups was always higher than in the non-supplemented group. At 120 hpt, the average count of live cells in the supplemented groups was 2–4 × 10^6^, while the count for non-supplemented cells was 1–2 × 10^6^. Similarly, the non-supplemented group’s live percentage dropped drastically after 96–120 hpt. Although the supplemented groups’ live percentage also dropped, the decline was gradual, and on harvest day, it was always higher than that of the non-supplemented samples. The total protein densitometric data obtained from stain-free blotting of 2-fold serial dilutions showed that 1/4, 1/8, and 1/16 dilutions of the TCA pellet were in the linear range (Supplementary Figure S2). The R-squared value was 0.99. Similarly, the densitometric data from blots developed with the fluorescent anti-His-tag antibody showed a linear range at 1/8 to 1/64 dilution, with an R-squared value of 0.98 (Supplementary Figure S3). Apart from the amount of sample loaded, the amount of antibody used can also lead to signal saturation or background noise. Therefore, the linear range was determined using a twofold dilution series of the Alexa_488_-labelled anti-His-tag antibody. The results indicate that antibody dilutions between 1:1000 and 1:8000 are in a linear range, with an R-squared value of 0.98 (Supplementary Figure S4). Overall, the analysis showed that hydrolysate-fed replicates have higher intensity bands (Figure 2). The protein expression levels from the two plant-derived hydrolysates were not significantly different from those of TN1. However, all three were better than having no hydrolysate (Figure 3 and Figure S5).

## 4. Discussion

The increased use of mammalian cells for the expression of recombinant proteins has led to the utilisation of less time-consuming transient transfections as compared with stable transfections. Recombinant protein production depends on several culture and transfection parameters, including cell density, DNA concentration, volume, temperature, harvesting time, serum addition or deprivation, and nutrient supplementation [27]. Supplementations like amino acids, glucose, and serum have been previously studied. Feeding of free amino acids has been shown to affect the yield negatively [18]. Although the serum-supplemented media have shown increased productivity [26], the high concentration of proteins in serum interferes with the downstream purification of secreted proteins [32], making this a less suitable option. A recent study investigated the effect of supplementation with casein, skimmed milk, casamino acid, amino acid mixtures (Tryptone, Biobasic), and an enzymatic digest of bovine and porcine animal products (Bacto Hydrolysate) by adding a 1 mg/mL concentration of the above to HEK293 cell cultures expressing an antibody on day 7. The study observed that only Bacto Hydrolysate could significantly boost the antibody production, while all the other supplements showed no significant difference as compared with no supplement control [27]. Among hydrolysates, the addition of TN1 has been shown to increase the expressed protein and is considered a standard supplement [26]. In addition, studies that explored the effect of direct supplementation of TN1, its impact on transfection efficiency, and the time of addition have also been conducted. TN1 has also been shown to completely inhibit the expression of recombinant protein when added at zero hpt. It has been suggested that hydrolysates increase recombinant protein by stimulating gene expression mostly during the post-transfection phase instead of the perceived effect on increasing the efficiency of transfection [26]. The impact of multiple feedings of TN1 has also been studied. Results indicate that a single feed between 24 and 48 hpt is sufficient, as additional feedings did not increase recombinant protein expression. TN1 was shown not to significantly increase growth but to increase yield. This may be because synthetic and proliferative processes require different nutritional blocks [26]; a similar phenomenon was also observed by [33].

Here, we compared the effect of two novel plant-based hydrolysates on the expression of a tegumental protein from the eukaryotic parasite *Echinococcus granulosus* in HEK293-6E cells with the standard TN1 hydrolysate. Transfection success and efficiency in the current study were monitored in parallel with GFP transfection. This was used as a quality control for the transfection reagents and the protocol used. Strictly speaking, a separate transfection with GFP would represent an inadequate transfection control, as this would be performed in parallel. Instead, it would have been necessary to track the uptake of a labelled plasmid, as suggested by Johnson et al., 1999 [34]. For these reasons, we chose not to assess the transfection efficiency in the individual 12 flasks, instead opting to use cells derived from a single transfection in a 1 L flask, subdivided into 12 equal parts. This rules out different efficiencies of transfection as a source of variability between replicates, as all cells were obtained from a single transfection.

As opposed to its commonly perceived use as a qualitative technique, Western blotting can be utilised as a quantitative tool when a stringent workflow is followed [35]. In case of overloading the gels, densitometry is not directly proportional to an increase in quantity [31]. Hence, we determined the linear dynamic range for protein loading by running a 2-fold serial dilution of the pooled sample, as described by [36] (Figure S2). Taylor et al. [36] showed that fluorescent antibodies have a broader linear range than a corresponding HRP-conjugated antibody using chemiluminescence-substrate-based detection. Therefore, we used an Alexa_488_-fluorophore-labelled anti-His-tag antibody to quantify the expressed protein. After all the optimisation steps, we observed that plant-derived hydrolysates yielded comparable recombinant protein.

We conclude that pea- or broad bean-derived hydrolysates, and possibly plant-derived hydrolysates more broadly, are a suitable replacement for TN1 in HEK293 recombinant protein expression. TN1 has been widely used, as it boosts recombinant protein yields by supplying readily utilisable peptides and amino acids that enhance cellular productivity; it promotes robust cell growth and viability, supporting high-density cultures commonly used in transient gene expression workflows. Furthermore, TN1 is easy to integrate into existing upstream processes due to its high solubility and well-documented performance across multiple mammalian expression platforms, including HEK293 and CHO cells. This is why it has become a standard component in many protocols [8,26]. As shown here, plant-derived hydrolysates perform equally well in terms of supporting growth and recombinant expression. However, it has the additional benefits of being more sustainable and less likely to be affected by import regulations than the use of animal-derived materials, and is therefore a promising alternative to TN1.

## Data Availability

The original contributions presented in this study are included in the article/Supplementary Material. Further inquiries can be directed to the corresponding author.

## Supplementary Materials

The following supporting information can be downloaded from https://www.mdpi.com/article/10.3390/biotech15010014/s1: Figure S1. Illustration of the workflow used in this study; Figure S2. Identification of the linear range for protein on stain-free blot; Figure S3. Linear range determination for the fluorescent antibody; Figure S4. Linear range for protein loading; Figure S5. Western blots for experimental repeats B and C.; Table S1. Trypan blue viability counts for days 1, 3 and 5.
