# Supplementary material for: Plant-Derived Hydrolysates Are a Suitable Replacement for Tryptone N1 in Recombinant Protein Expression Using Human Embryonic Kidney (HEK293-6E) Cells
Source: BioTech (Basel). 2026 Feb 5;15(1):14. doi: 10.3390/biotech15010014 (PMC12922121; doi:10.3390/biotech15010014)
Supplement: Supplementary file 1 [file biotech-15-00014-s001.zip › biotech-4012230-supplementary-after proof.pdf]

biotech-4012230

Supplementary Figures

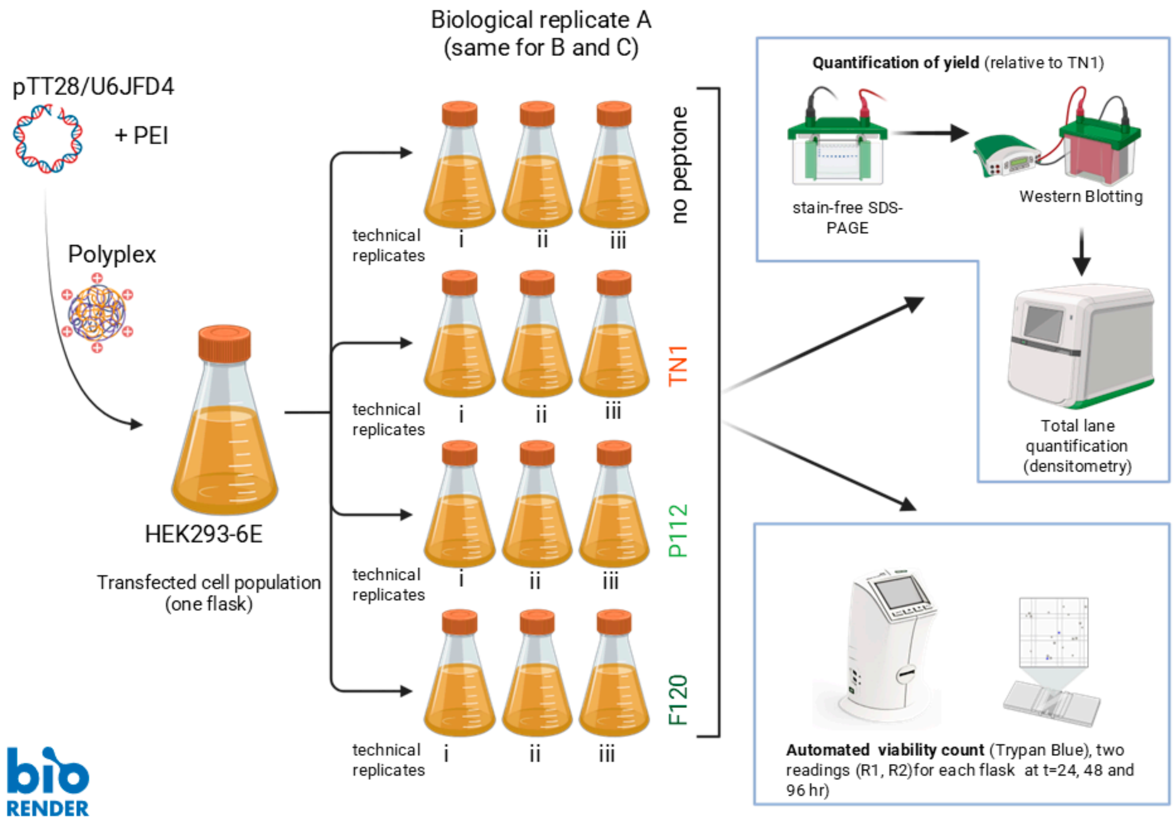

Figure S1. Illustration of the workflow used in this study. HEK293-6E cells were transfected with PEI-polyplexed pTT28 plasmid encoding the *Echinococcus granulosus* tegumental protein with the UniProt ID U6FJD4. The transfected cells were split equally into 12 125 mL flasks, which were supplemented with either no peptone or with TN1, P112, and F120 (technical triplicates i, ii, iii). Viability counts (two counts per time point/flask) were taken after 24, 96, and 120 hours. After 120 hours, supernatants were harvested and the yield of recombinant U6FJD4 protein quantified by stain-free densitometry. The whole experiment was repeated independently two more times (Biological repeats A, B, and C).

Fig S2: Determination of linear range on stain-free blot: Total Protein

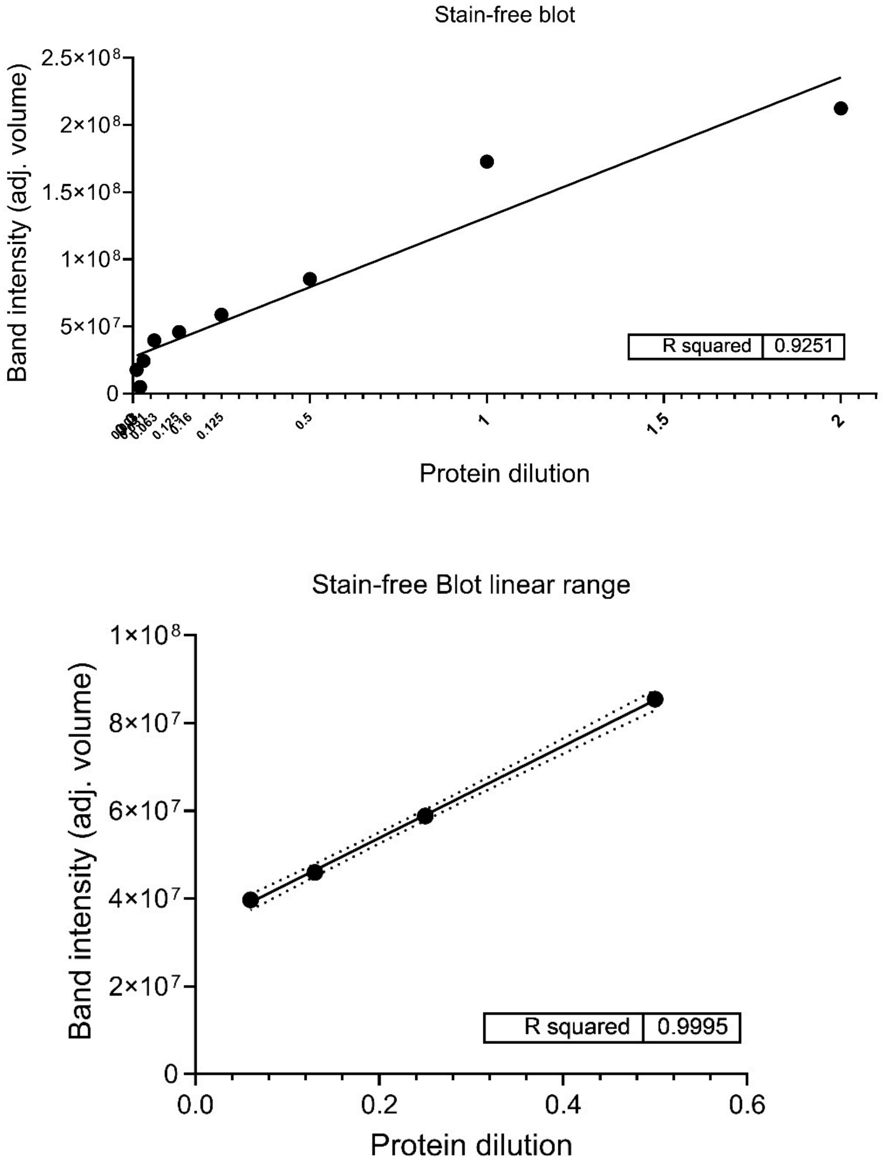

Figure S2. Identification of the linear range for protein on stain-free Blot. A 2-fold serial dilution series of the combined pooled sample (TCA-precipitation) was separated on a stain-free SDS-PAGE blot and transferred by blotting. **A:** Measured Band intensity (y-axis) vs. dilution factor. The R^2 value is 0.92. **B:** Section of graph B showing only those dilutions where R^2 value is >0.97, i.e., 1/4, 1/8, and 1/16 dilutions of the TCA pellet were in the linear range.

Fig S3: Determination of linear range for anti-His-tag-Alexa488 antibody developed Blot

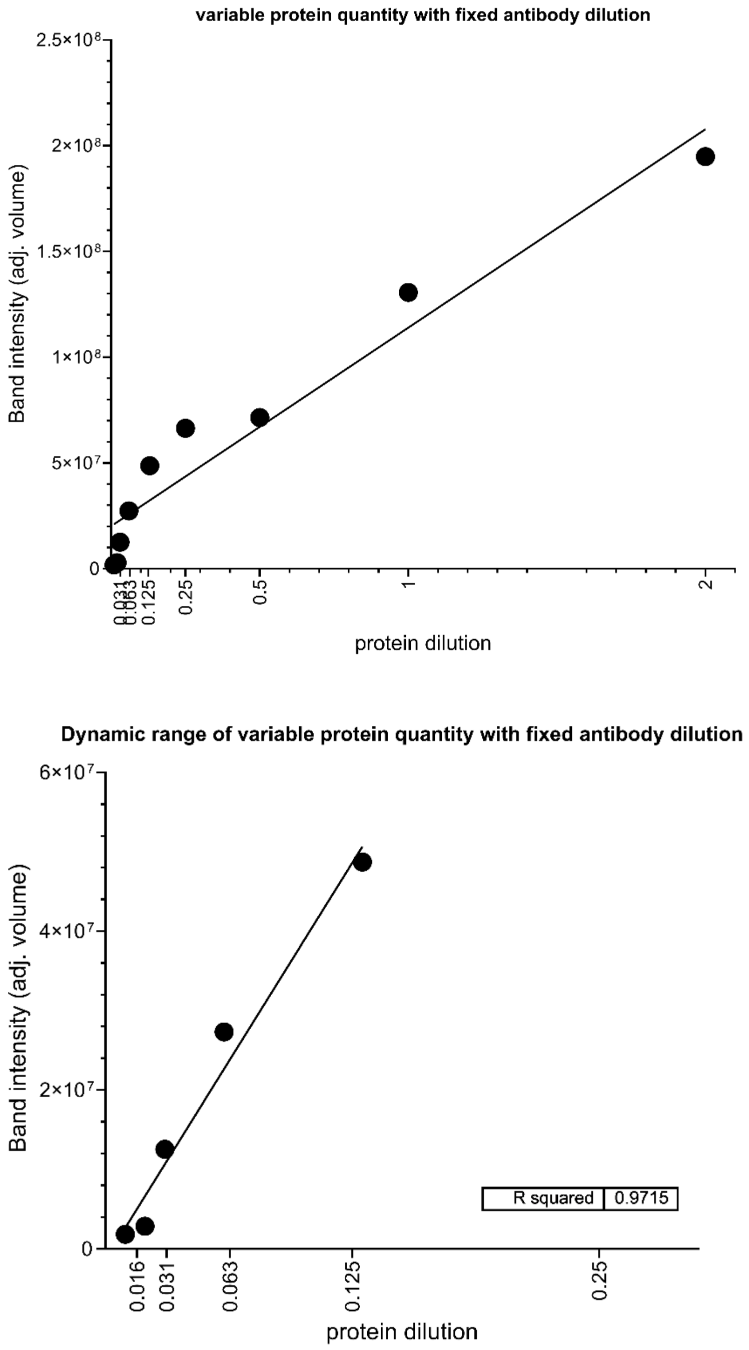

Figure S3. Western Blot developed with fluorescent antibody to investigate the linear range of protein loading with the antibody. Here, the antibody concentration was kept constant, while protein was loaded in 2-fold serial dilutions. **A:** Measured Band intensity (y-axis) vs. dilution factor. The R^2 value is 0.93. **B:** Section of graph B showing only those dilutions where R^2 value is >0.97, i.e., dilutions from 1/8 to 1/64.

Fig. S4: Determination of Linear Range for anti-His-tag Alexa488 antibody dilutions

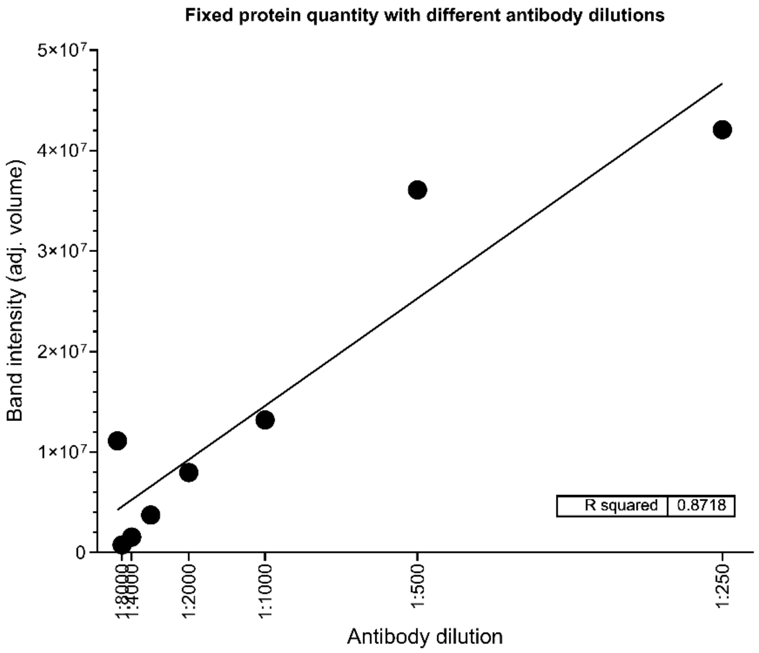

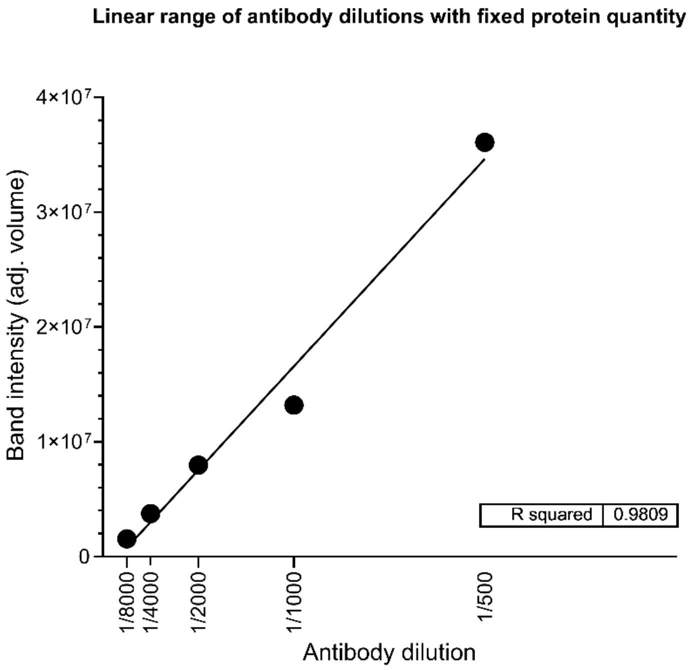

Figure S4. After determination of the linear range for protein loading, the linear range of antibody dilution was determined by loading 1/8 dilution of protein in each well in the stain-free gel and blotted after completion of the run. The membrane was cut into eight equal strips and each incubated with a different dilution of antibody, ranging from 1:250 (lane no.1) to 1:16000 (lane no.8). **A:** Graph developed by plotting the densitometric band intensity of fluorescence as the y-axis vs. the 2-fold dilutions of antibody as the x-axis, yielding an R^2 value of

0.87. **B:** Section of graph A showing only those dilutions where the R^2 value is >0.97.

Supplementary Figure S5: Biological Repeats B and C

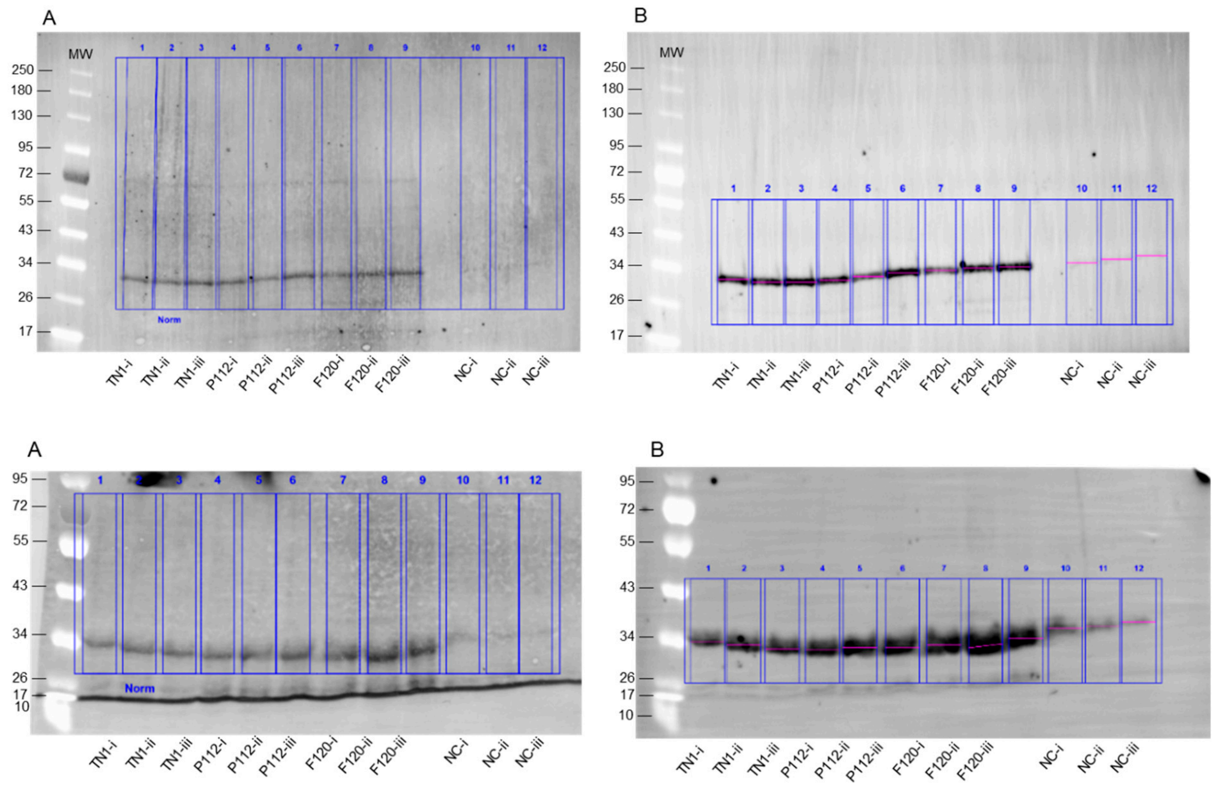

Figure S5. Western blots of the other two experimental repeats (B and C). **A:** Stain-free blot images of each experiment were taken before the development of the blot for normalization processes**. B:** Visualization of Western blots developed with an Anti-his-tag $Alexa_{488}$ fluorescent antibody and their quantifi
